# Safety Measures for Maintaining Low Endophthalmitis Rate after Intravitreal Anti-Vascular Endothelial Growth Factor Injection before and during the COVID-19 Pandemic

**DOI:** 10.3390/jcm11030876

**Published:** 2022-02-07

**Authors:** Koji Tanaka, Hiroyuki Shimada, Ryusaburo Mori, Yorihisa Kitagawa, Hajime Onoe, Kazuki Tamura, Hiroyuki Nakashizuka

**Affiliations:** Department of Ophthalmology, School of Medicine, Nihon University, 1-6 Surugadai, Kanda, Chiyodaku, Tokyo 101-8309, Japan; tanaka.koji@nihon-u.ac.jp (K.T.); ryu-m@sa2.so-net.ne.jp (R.M.); k.yorihisa@gmail.com (Y.K.); onoe.hajime@nihon-u.ac.jp (H.O.); tamura.kazuki@nihon-u.ac.jp (K.T.); nkshizuk@gmail.com (H.N.)

**Keywords:** adhesive face drape, COVID-19 pandemic, endophthalmitis, intravitreal injection, vascular endothelial growth factor, 0.25% povidone-iodine

## Abstract

During the COVID-19 pandemic, intravitreal injections are performed with patients wearing masks. The risk of endophthalmitis after intravitreal injection is reported to increase due to an influx of exhaled air containing oral bacteria from the upper part of the mask onto the ocular surface. We retrospectively investigated the incidence of endophthalmitis when intravitreal anti-vascular endothelial growth factor (anti-VEGF) injections were performed using the same infection control measures before and during the pandemic. Vitreoretinal specialists performed intravitreal injections of anti-VEGF agents in the outpatient room of a university hospital. Infection control measures before and during the pandemic included covering the patient’s eye with adhesive face drape and irrigating the ocular surface with 0.25% povidone-iodine before draping, and immediately before and after injection. Before the COVID-19 pandemic (February 2016 to December 2019), one case of endophthalmitis occurred among 31,173 injections performed (0.0032%; 95% confidence interval (CI), 0.000008–0.017872%). During the COVID-19 pandemic (January 2020 to August 2021), one case of endophthalmitis occurred among 14,725 injections performed (0.0068%; 95% CI, 0.000017–0.037832%). There was no significant difference between the two periods (Fisher’s exact test: *p* = 0.5387). Even during the COVID-19 pandemic, very low incidence of endophthalmitis after intravitreal injection can be maintained by implementing basic infection prophylactic measures, including face draping and 0.25% povidone-iodine irrigation, established before COVID-19 pandemic.

## 1. Introduction 

Intravitreal injection of anti-vascular endothelial growth factor (VEGF) is the most widely used treatment for various retinal disorders [1]. The incidence of endophthalmitis after intravitreal injection calculated from the data reported by four large studies was 1144/2,394,429 (0.048%) (95% confidence interval (CI): 0.045–0.0505%) [2,3,4,5]. The endophthalmitis rates differ depending on the underlying diseases. Topical administration of antibiotics before and after intravitreal injection is not practiced in many countries, because this practice does not reduce the incidence of endophthalmitis, and also increases antibiotic resistance [6,7,8,9]. 

In 2014, guidelines for intravitreal injection were reported by American experts [10]. Endophthalmitis associated with intravitreal injection occurs when the conjunctival flora or the oral flora (such as *Streptococcus* species) of the patient and/or the physician are carried into the vitreous by the injection needle [11,12,13]. Effective prophylactic measures against endophthalmitis include implementing a no-talking policy or the use of face masks by patients and physicians, avoiding eyelid contact with injection site and needle, and applying povidone-iodine [14,15,16].

Since the beginning of the COVID-19 pandemic, patients, physicians, and hospital staff routinely wear face masks [17,18,19]. However, studies have found that after wearing a face mask for more than four hours, the periocular area becomes contaminated with the oral flora, which can spread from the top of the mask to the eye surface, posing a risk for endophthalmitis [20,21,22]. Measures, such as securing the top edge of the face mask with tape, have been reported to prevent the air jet containing bacteria from spreading in the direction of the eye [22,23,24]. Three recent articles compared the incidence of endophthalmitis after intravitreal injections during the COVID-19 pandemic (with implementation of patient masking) with its incidence in the pre-COVID-19 era [25,26,27]. Data show that, when measures such as taping the upper edge of the face mask were used, the incidence of endophthalmitis was 131/434,690 (0.030%) before the COVID-19 pandemic and 59/248,510 (0.024%) during the COVID-19 pandemic, with no significant difference. These reports indicate that taping the upper edge of a patient’s mask during the COVID-19 epidemic did not improve the incidence of endophthalmitis compared to before the COVID-19 pandemic [25,26,27].

Before the COVID-19 pandemic, the authors reported the usefulness of routine infection prophylaxis measures, including face draping and ocular surface irrigation with 0.25% povidone-iodine during intravitreal injection of anti-VEGF agents, in minimizing post-injection endophthalmitis [8,28]. However, whether these measures are effective in preventing endophthalmitis during the COVID-19 pandemic has not been reported. We therefore compared the post-injection endophthalmitis rates before and during the COVID-19 pandemic, when the same basic standardized prophylactic measures were implemented in both periods. 

## 2. Methods

### 2.1. Study Design and Study Population

In this retrospective, single-center, comparative cohort study, we investigated the incidence of endophthalmitis after intravitreal injection of anti-VEGF agents. Patients who received intravitreal injections of anti-VEGF agents at Nihon University Hospital between February 2016 and August 2021 were identified from medical records. Intravitreal injections of aflibercept, ranibizumab, brolucizumab, pegaptanib, and bevacizumab were included in the analysis. Intravitreal injections of triamcinolone acetonide were excluded following the selection criteria used in the authors’ previous study on the frequency of endophthalmitis after intravitreal injections [8]. The indications for treatment included age-related macular degeneration, polypoidal choroidal vasculopathy, retinal angiomatous proliferation, macula edema with retinal vein occlusion, diabetic macular edema, myopic choroidal neovascularization, and proliferative diabetic retinopathy.

### 2.2. Intravitreal Injection Technique

Basic infection prophylactic measures focused on two protocols. First, the patient’s eye was covered with a sterile adhesive face drape. Second, the ocular surface was irrigated with 0.25% povidone-iodine before draping, immediately before and immediately after the injection. Two periods were studied: (1) Before the COVID-19 pandemic, when patients were not wearing face masks, and the eyes were covered with an adhesive face drape after disinfection of the ocular surface; (2) During the COVID-19 pandemic, when patients were wearing face masks, the mask was pulled downward to the chin, and the eye was covered with an adhesive face drape after disinfection of the ocular surface.

**Figure 1:**
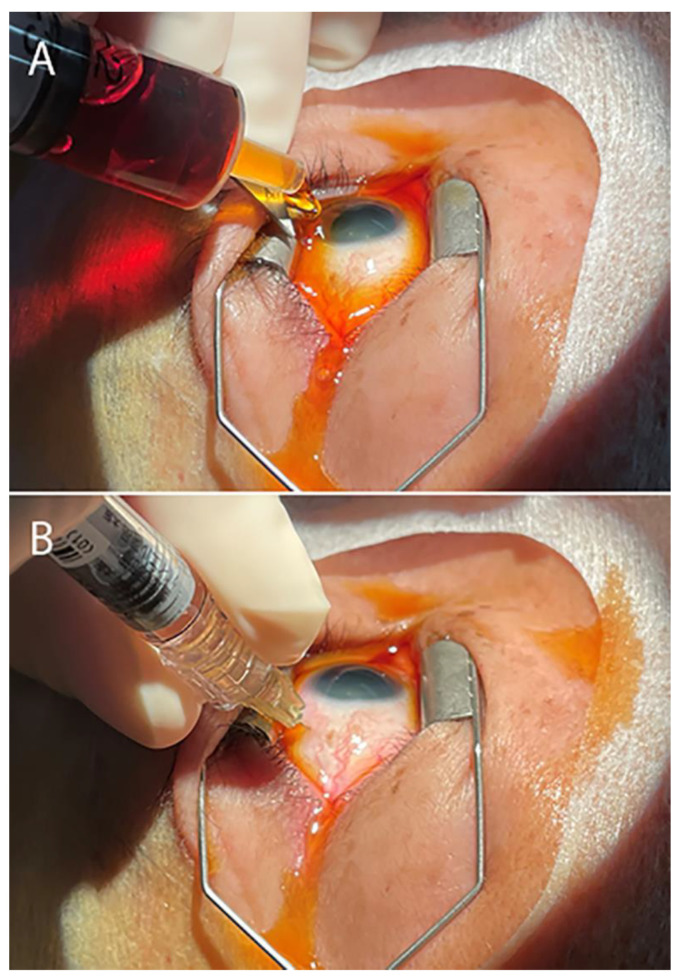
Intravitreal injection using face drape and 0.25% povidone-iodine irrigation during the COVID-19 pandemic. (**A**) After disinfecting the eyelid skin and ocular surface with at least 10 mL of 0.25% povidone-iodine, the eye was covered with an adhesive face drape, and a solid blade lid speculum was placed, then the eye surface was irrigated again with several ml of povidone-iodine. (**B**) After waiting for 30 s, intravitreal injection was performed while povidone-iodine remained on the ocular surface. After injection, the ocular surface was irrigated again with several ml of povidone-iodine to complete the procedure.

In both periods, nurses in the outpatient room diluted 10% povidone-iodine (Shionogi Pharmaceutical, Osaka, Japan) in saline to prepare a 0.25% povidone-iodine solution [29]. Intravitreal anti-VEGF injections were performed by vitreoretinal specialists in the outpatient room, which is separated from the outpatient clinic and equipped with one microscope and one bed. Physicians and nurses wore white coats, caps, and surgical masks. The caps and surgical masks were changed twice a day (morning and afternoon). To prepare for injection, the eye was instilled with topical anesthetic and dilating agents. The patient wore a cap and lay on the bed with his/her own clothes without taking off the shoes. Before injection, the physician disinfected the eyelids, eyelashes, and conjunctival fornix with at least 10 mL of 0.25% povidone-iodine. Using sterile gloves, a sterile adhesive face drape was applied to cover the eye. A sterile solid blade lid speculum was applied, and 4% xylocaine was instilled. The conjunctiva was irrigated with several ml of 0.25% povidone-iodine (Figure 1A). After waiting for 30 s while preparing the anti-VEGF agent, intravitreal injection was started while povidone-iodine remained on the ocular surface (Figure 1B). Intravitreal injection was performed 4 mm from the limbus measured by a caliper. Finally, the ocular surface was irrigated with several ml 0.25% povidone-iodine and hand movement was confirmed. After injection, no eye patch was worn. The patient was educated about the symptoms of endophthalmitis and the need for immediate consultation should any symptom arise. Appropriate follow-up appointments were scheduled. Patients were monitored for at least 3 months after intravitreal injection for the occurrence of endophthalmitis.

### 2.3. Statistical Analysis

Statistical analysis of the data was performed using SPSS software for Windows, version 12 (Chicago, IL, USA). Statistical significance was set at *p* < 0.05. Data obtained from the study population is expressed as a percentage (95% CI). Fisher’s exact test was used to compare the two groups. 

## 3. Results

A total of 45,898 intravitreal injections of anti-VEGF agents were performed in 8609 eyes. The indications for intravitreal injection of anti-VEGF agents were age-related macular degeneration, polypoidal choroidal vasculopathy and retinal angiomatous proliferation (together 79% of all eyes), macula edema with branch retinal vein occlusion (8%), diabetic macular edema (7%), myopic choroidal neovascularization (2%), macula edema with central retinal vein occlusion (1%), proliferative diabetic retinopathy (1%), and others (2%).

### 3.1. Incidence of Endophthalmitis

Prior to the COVID-19 pandemic, a total of 31,173 intravitreal injections were performed between February 2016 and December 2019. Of these, one case of proven infectious endophthalmitis occurred (1/31,173 = 0.0032%; 95% confidence interval (CI), 0.000008–0.017872) (Table 1).

During the COVID-19 pandemic, a total of 14,725 intravitreal injections were performed between January 2020 and August 2021. Of these, one case of proven infectious endophthalmitis occurred (1/14,725 = 0.0068%; 95% CI, 0.000017–0.037832%). No significant difference in the incidence of endophthalmitis was observed between the two periods (Fisher’s exact test: *p* = 0.5354). 

The anti-VEGF agents and the numbers of injections are summarized in Table 1. Prior to the COVID-19 pandemic, a total of 31,173 injections comprising 23,666 injections of aflibercept, 6945 injections of ranibizumab, 0 injections of brolucizumab, 134 injections of pegaptanib, and 428 injections of bevacizumab were performed. During the COVID-19 pandemic, a total of 14,725 injections, comprising 11,829 injections of aflibercept, 2308 injections of ranibizumab, 386 injections of brolucizumab, 6 injections of pegaptanib, and 196 injections of bevacizumab were performed. 

### 3.2. Cases of Endophthalmitis

Case prior to the COVID-19 pandemic. A 75-year-old man developed endophthalmitis on the third day after the fourth intravitreal injection of aflibercept in his left eye for age-related macular degeneration. His visual acuity was 4/20 at the fourth intravitreal injection, and had movement when presenting with endophthalmitis. Emergency vitrectomy was performed, and his corrected visual acuity at 3 months after operation was 8/20. The causative organism was *Morganella morganii*, a Gram-negative rod belonging to the family *Enterobacteriaceae*.

Case during the COVID-19 pandemic. An 82-year-old woman developed endophthalmitis on the second day after the third intravitreal injection of aflibercept in her right eye for age-related macular degeneration. Her visual acuity was 8/20 at the third intravitreal injection and was finger counting when presenting with endophthalmitis. Emergency vitrectomy was performed and her corrected visual acuity at 3 months after operation was 10/20. The causative organism was *Staphylococcus epidermidis*.

## 4. Discussion

Prior to the COVID-19 pandemic, a meta-analysis of endophthalmitis after intravitreal anti-VEGF injection reported a significantly higher rate of *Streptococcus* spp. in culture-positive cases (30.8%) compared to cataract surgery (8.2%) or vitrectomy (9.0%), suggesting a need to minimize oropharyngeal droplet transmission by wearing surgical masks and avoiding talking during intravitreal injection [13]. However, since the beginning of the pandemic, patients, doctors, and hospital staff have routinely worn face masks. Does this situation have an impact on the organisms causing intraocular infection? In the three reports mentioned in the Introduction [25,26,27], the percentage of oral flora in culture-positive cases was lower during the COVID-19 pandemic (5.9%) than before the pandemic (16.7%), but the differences were not significant due to the small number of cases. The percentage of oral flora infections could have been reduced during the pandemic by measures such as taping the upper edge of the patient’s face mask. This point needs further verification.

Before the COVID-19 pandemic, there was no evidence to support routine use of drapes when performing intravitreal injections [10]. During the pandemic, while taping the top edge of the face mask may be effective, depending on the shape of the face, this practice may not reliably stop airflow towards the ocular surface [23]. We believe that adhesive face drapes that completely block the patient’s mouth and ocular surface are necessary to reduce the incidence of endophthalmitis following intravitreal injection during the COVID-19 pandemic. 

The 2014 guidelines recommend that care should be taken to ensure that the tip of the needle does not touch the eyelid or eyelashes during intravitreal injection [10]. Indeed, the normal flora load in the eye is known to be higher at the eyelid margin than at the conjunctiva [30]. However, use of a lid speculum prior to intraocular injection does not affect the number of bacteria on the conjunctiva [31], presumably due to the flowing of the normal flora between the wire lid speculum onto the ocular surface. We used a solid blade lid speculum instead of the usual wire lid speculum to prevent the needle tip from touching the eyelid or eyelashes.

We believe that povidone-iodine is the most important measure for preventing endophthalmitis after intravitreal injection. When using povidone-iodine, it is important to understand the optimal concentration used, the contact time required, and the amount and frequency of application [32,33,34]. 

The 2014 guidelines recommend performing intravitreal injection approximately 30 s after applying povidone-iodine on the ocular surface [10]. Therefore, it is necessary to select a concentration that exhibits a rapid bactericidal effect. The most effective concentration of povidone-iodine is 0.1%, which exhibits a bactericidal effect within 15 s of exposure [34,35]. Since povidone-iodine is diluted when applied to the ocular surface, use of a concentration slightly higher than 0.1% is recommended. We have been using 0.25% povidone-iodine [8,28]. Povidone-iodine is inactivated when it acts on bacteria, and 0.25% povidone-iodine with a low iodine content does not remain effective for long after a single irrigation [32,33]. Hence, we irrigate the ocular surface twice before injection to maintain the disinfection effect. Povidone-iodine use at concentrations higher than 0.1% has been reported, including 0.3% povidone-iodine [36], 0.625–2.5% povidone-iodine [37], and a commercially available preparation of 0.6% povidone-iodine [38]. Five percent povidone-iodine with a high iodine content maintains the disinfecting effect for a long time after a single application, but it requires a long exposure time of 120–180 s to exhibit a bactericidal effect [35]. However, the 5% povidone-iodine ophthalmic solution, when diluted 5–10 times on the ocular surface, becomes 0.5–1.0%, a concentration that requires contact time of around 30 s [35], which satisfies the recommendation of the 2014 guidelines [10].

As for the volume of povidone-iodine to be used, studies have shown that irrigating the ocular surface with 10 mL of 5% povidone-iodine achieved a significantly greater reduction in conjunctival bacterial flora compared with application of 2–3 drops of povidone-iodine before surgery [39,40]. The conjunctiva in the fornix has many deep crypts. A reasonable assumption is that povidone-iodine reaches the fornix and the surface of these crypts much more effectively after mechanical irrigation than after application of 2–3 drops on the bulbar conjunctiva [39]. For this reason, we disinfect the eyelids, eyelashes, and conjunctival fornix with at least 10 mL of 0.25% povidone-iodine before draping; irrigate the ocular surface with several ml of povidone-iodine after placing the solid blade lid speculum; wait for 30 s; and then perform intravitreal injection while the povidone-iodine remains on the ocular surface. According to a study performed using rabbit eyes, there is no retinal damage associated with washing the ocular surface with 0.25% povidone-iodine during intravitreal injection [41]. After injection, the ocular surface is again disinfected with several ml of povidone-iodine to prevent endophthalmitis [42].

Universal face masking, the practice of face mask use by physicians, hospital staff, and patients, has been adopted as a prophylaxis measure for intravitreal injections during the COVID-19 pandemic. A multicenter large-scale retrospective study found that universal face mask use during intravitreal injections did not alter the risk of post-injection endophthalmitis [27]. In addition, taping the top of the patient’s mask also had no significant effect. Our study showed that using an adhesive face drape instead of a mask with taping for the patient, together with routine prophylactic measures, is equally effective to maintain a low incidence of endophthalmitis.

The major limitations of this study were that it was underpowered because the sample size was small, especially when compared to the large-scale study by Patel et al. [27], and that it was a single-center, retrospective study. Further large-scale observation is needed to improve the statistical power of the study. Further, there was no control group that did not use face draping and 0.25% povidone-iodine irrigation in this study. A meta-analysis may reveal whether the organisms causing endophthalmitis differ before the COVID-19 pandemic and during the pandemic. In the present study, there was a change in the distribution of anti-VEGF agents used before and after starting of the COVID-19 pandemic. However, another study showed a similar incidence of endophthalmitis after intravitreal injection of bevacizumab, ranibizumab, and aflibercept [43]. Although the incidence of endophthalmitis after intravitreal injection of anti-VEGF drugs is low, the prognosis is unfavorable [2,3,4,5]. Further prevention strategies should be sought. The authors of this study believe that using adhesive face draping is more effective for preventing the spread of the patient’s oral bacteria to the ocular surface than taping the superior portion of a patient’s face mask, and, hence, may impact the post-injection endophthalmitis rate.

In conclusion, the incidence of endophthalmitis during the COVID-19 pandemic can be maintained at a very low rate by implementing the same basic infection prophylactic measures, including face draping and 0.25% povidone-iodine ocular surface irrigation, established before the COVID-19 pandemic.

## Figures and Tables

**Table 1:** Anti-VEGF Type and Numbers of Intravitreal Injections in Pre-COVID-19 and During COVID-19 Pandemic.

	Number of Intravitreal Injections for Each Anti-VEGF Agent					Incidence of Endophthalmitis *	95% Confidence Interval
	Aflibercept	Ranibizumab	Brolucizumab	Pegaptanib	Bevacizumab		
Pre-COVID-19 pandemic	23,666	6945	0	134	428	1/31,173 0.0032%	0.000008–0.017872%
During COVID-19 pandemic	11,829	2308	386	6	196	1/14,725 0.0068%	0.000017–0.037832%

VEGF: vascular endothelial growth factor, COVID-19: coronavirus disease-2019; * Fisher’s exact test (two-sided); pre-COVID-19 vs during COVID-19 pandemic: *p* = 0.5387.
